# Consequences of group III/IV afferent feedback and respiratory muscle work on exercise tolerance in heart failure with reduced ejection fraction

**DOI:** 10.1113/EP090755

**Published:** 2023-09-21

**Authors:** Joshua R. Smith, Jonathon W. Senefeld, Kathryn F. Larson, Michael J. Joyner

**Affiliations:** 1 Department of Cardiovascular Medicine, Mayo Clinic, Rochester, MN, USA; 2 Department of Anesthesiology and Perioperative Medicine, Mayo Clinic, Rochester, MN, USA; 3 Department of Kinesiology and Community Health, University of Illinois at Urbana-Champaign, Urbana, IL, USA

**Keywords:** diaphragm, exercise pressor reflex, metaboreflex, respiratory muscle blood flow

## Abstract

Exercise intolerance and exertional dyspnoea are the cardinal symptoms of heart failure with reduced ejection fraction (HFrEF). In HFrEF, abnormal autonomic and cardiopulmonary responses arising from locomotor muscle group III/IV afferent feedback is one of the primary mechanisms contributing to exercise intolerance. HFrEF patients also have pulmonary system and respiratory muscle abnormalities that impair exercise tolerance. Thus, the primary impetus for this review was to describe the mechanistic consequences of locomotor muscle group III/IV afferent feedback and respiratory muscle work in HFrEF. To address this, we first discuss the abnormal autonomic and cardiopulmonary responses mediated by locomotor muscle afferent feedback in HFrEF. Next, we outline how respiratory muscle work impairs exercise tolerance in HFrEF through its effects on locomotor muscle O_2_ delivery. We then discuss the direct and indirect evidence supporting an interaction between locomotor muscle group III/IV afferent feedback and respiratory muscle work during exercise in HFrEF. Last, we outline future research directions related to locomotor and respiratory muscle abnormalities to progress the field forward in understanding the pathophysiology of exercise intolerance in HFrEF.

## 1 | INTRODUCTION

Exercise training is a cornerstone therapy for cardiovascular disease, including patients with heart failure (HF). Exercise training is associated with improvements in exercise capacity and quality of life as well as reductions in mortality risk in HF patients (Flynn et al., Exercise training is a cornerstone therapy for cardiovascular disease, including patients with heart failure (HF). Exercise training is associated with improvements in exercise capacity and quality of life as well as reductions in mortality risk in HF patients (Flynn et al., 2009; O’Connor et al., 2009; Smart & Marwick, 2004). Despite the importance of exercise training, many patients with HF do not regularly engage in exercise due, in part, to physical symptomology limiting exercise tolerance (Piepoli et al., 2011; van der Wal et al., 2006). Although much is known about the epidemiology and aetiology of HF, this knowledge has yet to be integrated into a complete picture of physical symptomology in humans particularly during exercise. In this context, our review highlights the physiological responses arising from locomotor muscle group III/IV afferent feedback and respiratory muscle work as well as proposes that the concomitant effects of locomotor muscle afferent feedback and respiratory muscle work worsen exercise tolerance and exacerbate exertional dyspnoea in patients with HF. O’Connor et al., 2009; Smart & Marwick, 2004). Despite the importance of exercise training, many patients with HF do not regularly engage in exercise due, in part, to physical symptomology limiting exercise tolerance (Piepoli et al., 2011; van der Wal et al., 2006). Although much is known about the epidemiology and aetiology of HF, this knowledge has yet to be integrated into a complete picture of physical symptomology in humans particularly during exercise. In this context, our review highlights the physiological responses arising from locomotor muscle group III/IV afferent feedback and respiratory muscle work as well as proposes that the concomitant effects of locomotor muscle afferent feedback and respiratory muscle work worsen exercise tolerance and exacerbate exertional dyspnoea in patients with HF.

Current estimates suggest that nearly 6.5 million Americans are diagnosed with HF, and HF with reduced ejection fraction (HFrEF) accounts for ~50% of those diagnoses (Tsao et al., 2022). One of the cardinal symptoms of HF is exercise intolerance with HF patients having severely impaired peak oxygen uptake $\left({\dot{V}}_{O_{2}\text{peak}}\right)$, which is associated with higher mortality and morbidity rates (Francis et al., 2000), which is associated with higher mortality and morbidity rates (Francis et al., 2000; Malhotra et al., 2016; Mancini et al., 1991). However, resting measures of cardiac function (e.g., left ventricular ejection fraction) are not correlated with exercise capacity in HF patients (Coats, 2001; Franciosa et al., 1981). To better understand the underlying mechanisms of exertional dyspnoea and exercise intolerance in HF patients, Coats et al. put forth the ‘muscle hypothesis’ (Coats et al., 1994). The ‘muscle hypothesis’ suggests that skeletal muscle abnormalities including muscle atrophy, reduced capillary density, mitochondrial dysfunction, release of proinflammatory cytokines, and increased synthesis of reactive oxygen species contribute to the cardinal symptoms of HF (Aimo et al., 2021; Coats et al., 1994). One of the primary mechanisms by which the ‘muscle hypothesis’ and associated skeletal muscle abnormalities contribute to exercise intolerance and exertional dyspnoea in HF is through alterations in locomotor muscle group III/IV afferent feedback (Angius & Crisafulli, 2020; Smith, Joyner et al., 2020). Malhotra et al., 2016; Mancini et al., 1991). However, resting measures of cardiac function (e.g., left ventricular ejection fraction) are not correlated with exercise capacity in HF patients (Coats, 2001; Franciosa et al., 1981). To better understand the underlying mechanisms of exertional dyspnoea and exercise intolerance in HF patients, Coats et al. put forth the ‘muscle hypothesis’ (Coats et al., 1994). The ‘muscle hypothesis’ suggests that skeletal muscle abnormalities including muscle atrophy, reduced capillary density, mitochondrial dysfunction, release of proinflammatory cytokines, and increased synthesis of reactive oxygen species contribute to the cardinal symptoms of HF (Aimo et al., 2021; Coats et al., 1994). One of the primary mechanisms by which the ‘muscle hypothesis’ and associated skeletal muscle abnormalities contribute to exercise intolerance and exertional dyspnoea in HF is through alterations in locomotor muscle group III/IV afferent feedback (Angius & Crisafulli, 2020; Smith, Joyner et al., 2020).

**FIGURE 1:**
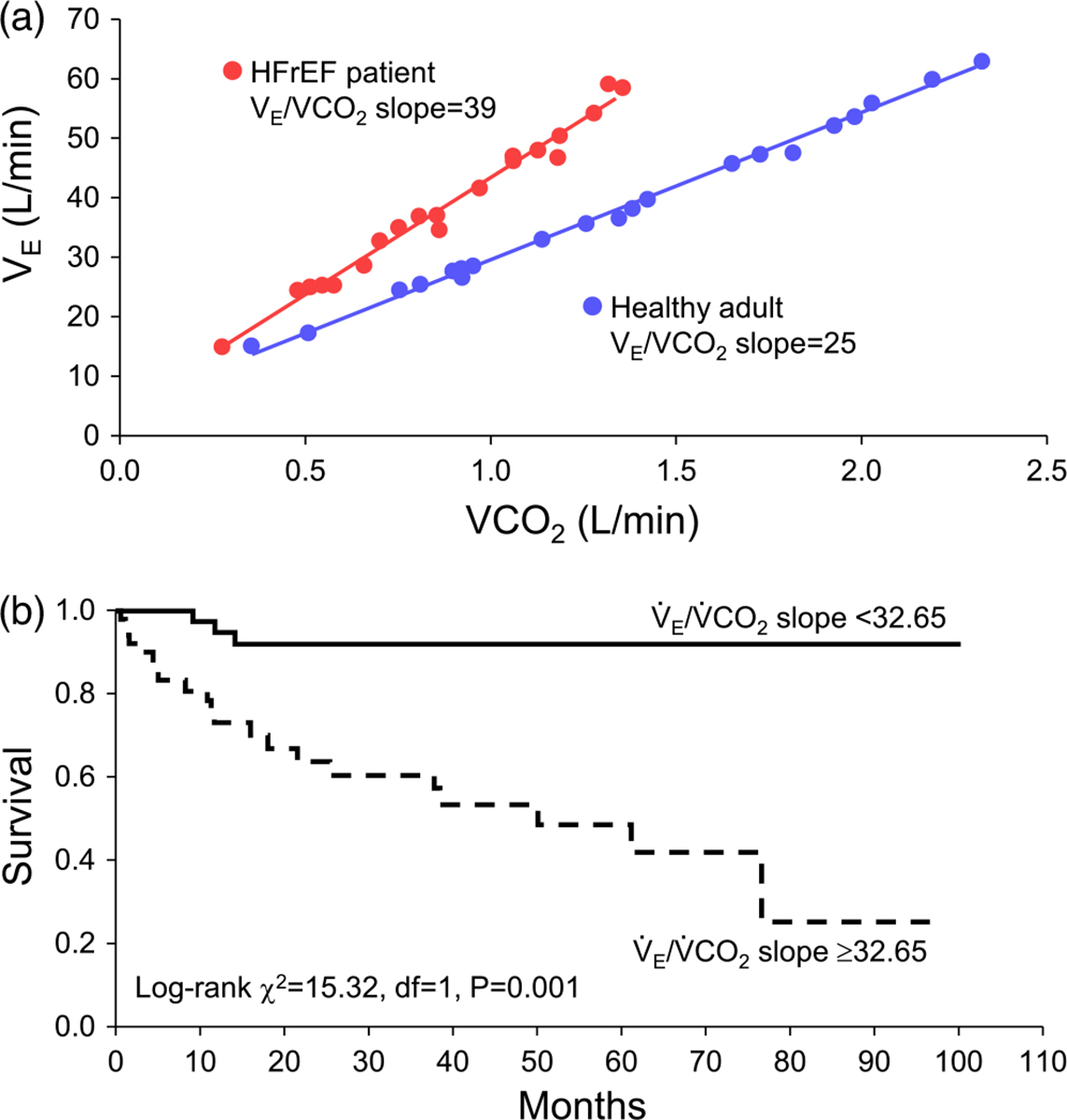
Ventilatory response in HFrEF and its impact on mortality. (a) The ventilation to carbon dioxide $\left({\dot{V}}_{E}/{\dot{V}}_{{CO}_{2}})\right.$ slope for a representative healthy control adult and HFrEF patient during incremental exercise. (b) The impact of slope for a representative healthy control adult and HFrEF patient during incremental exercise. (b) The impact of slope on mortality. A higher slope on mortality. A higher slope (i.e., ≥32.65 in this study) was significantly associated with lower survival compared to a lower ${\dot{V}}_{E}/{\dot{V}}_{{CO}_{2}}$ slope (<32.65) in patients with HFrEF. Data used with permission and adapted from Guazzi et al. (2005).

Patients with HFrEF also have pulmonary system and respiratory muscle abnormalities that negatively influence exercise tolerance. For example, HFrEF patients demonstrate an exaggerated ventilatory response to exercise (often reported as increased ventilation to carbon dioxide $\left({\dot{V}}_{E}/{\dot{V}}_{{CO}_{2}})\right.$ slope) (see Figure 1) (Dube et al., 2016). This exaggerated ventilatory response during exercise is associated with mortality in HFrEF and may have higher prognostic value than ${\dot{V}}_{O_{2}\text{peak}}$ in HFrEF (Arena et al., 2004). The disproportionate ventilatory response coupled with pulmonary system abnormalities and subsequently elevated respiratory muscle work plays an important role in the pathophysiology of exercise tolerance in HFrEF patients (Borghi-Silva et al., 2008; O’Donnell et al., 1999). In this context, one of the principal contributors to the pathophysiological ventilatory response in HFrEF is locomotor muscle afferent feedback (Aimo et al., 2021; Olson et al., 2014; Smith et al., 2022). Thus, the primary motivation for this review is to describe the integrated physiological response to exercise arising from locomotor muscle group III/IV afferent feedback and respiratory muscle work, as exemplified by the locomotor muscle afferent-mediated increase in ventilation.

## 2 | IMPACT OF LOCOMOTOR MUSCLE GROUP III/IV AFFERENT FEEDBACK ON CARDIOPULMONARY RESPONSES IN HFrEF

Autonomic adjustments to exercise are mediated by central command and the exercise pressor reflex, and also modulated by the arterial baroreceptors (Fisher et al., 2015; Mitchell et al., 1983; Smith et al., 2019). These autonomic adjustments are critical for the appropriate cardiovascular response to exercise including the increases in heart rate and blood pressure. The exercise pressor reflex is a feedback mechanism arising from the contracting skeletal muscles with the afferent arm comprising group III (predominantly mechanically sensitive) and group IV (predominantly metabolically sensitive) afferents that evokes sympathoexcitation and cardiovascular responses to exercise (Fisher et al., 2015; Mitchell et al., 1983). Locomotor muscle group III/IV afferent feedback is necessary for normal cardiovascular function during exercise in healthy adults. For example, inhibition of *μ*-opioid receptor sensitive group III/IV muscle afferent feedback via lower lumbar intrathecal fentanyl attenuates the increases in cardiac output, blood pressure, leg vascular conductance and locomotor muscle blood flow during exercise in healthy young adults (Amann et al., 2010; Amann, Blain et al., 2011; Amann, Runnels et al., 2011). Recent studies provide evidence that healthy ageing impacts the contribution of the locomotor muscle afferents to the central and peripheral haemodynamic response to exercise (Amann et al., 2014; Sidhu et al., 2015; Smith, Joyner et al., 2020; Smith et al., 2022). Specifically, the reduction in cardiac output during exercise with intrathecal fentanyl administration is smaller in older (~5%) compared to younger adults (17%) (Sidhu et al., 2015). Further, locomotor muscle afferent inhibition with fentanyl was associated with a decrease in blood pressure in the older adults (to a similar extent as the younger adults), but an increase in leg vascular conductance resulting in no effect on locomotor muscle blood flow compared to the placebo condition during exercise (Sidhu et al., 2015). These findings are important in the context of this review because HF disproportionally afflicts older adults (Tsao et al., 2022). In HFrEF, abnormal neural and cardiovascular responses arising from locomotor muscle group III/IV afferent feedback have been proposed to be central mechanisms impairing exercise capacity in these patients (Angius & Crisafulli, 2020; Smith, Joyner et al., 2020).

### 2.1 | Impact of locomotor muscle group III/IV afferent feedback on cardiopulmonary responses in HFrEF

**FIGURE 2:**
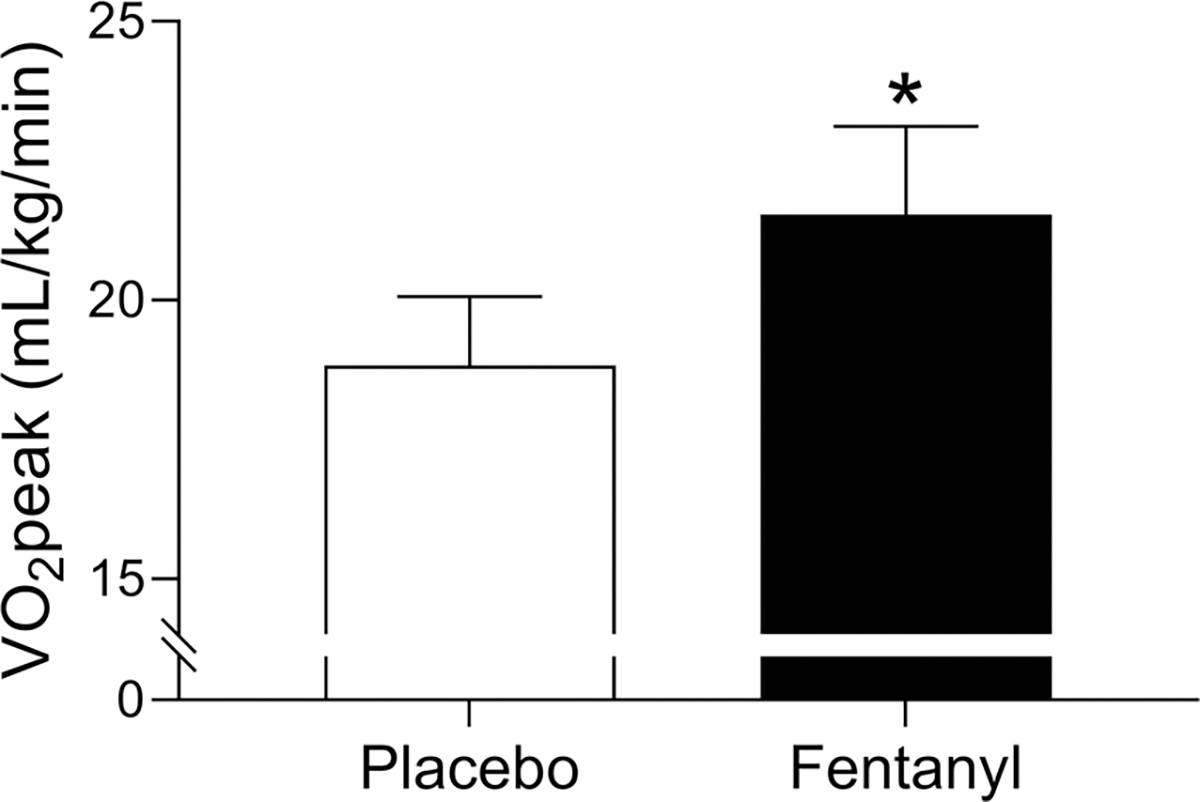
Impact of lower lumbar intrathecal fentanyl on ${\dot{V}}_{O_{2}\text{peak}}$ in HFrEF patients. ${\dot{V}}_{O_{2}\text{peak}}$ was 15% higher with fentanyl compared to placebo in patients with HFrEF. *Significantly different from placebo condition. Data used with permission and adapted from Smith, Joyner et al. (2020).

We recently investigated the notion that locomotor muscle group III/IV afferents contribute to exercise intolerance in patients with HFrEF. We investigated this by having healthy older control participants and HFrEF patients perform an exercise cycling test to measure peak workload and ${\dot{V}}_{O_{2}\text{peak}}$ with and without inhibition of locomotor muscle afferent feedback via lower lumbar intrathecal fentanyl (Smith, Joyner et al., 2020). Healthy older control participants had no change in peak workload and ${\dot{V}}_{O_{2}\text{peak}}$ between fentanyl and placebo conditions. In contrast, ${\dot{V}}_{O_{2}\text{peak}}$ and peak workload increased by 15 and 18%, respectively, with fentanyl compared to placebo condition in the HFrEF patients (see Figure 2). This magnitude of ${\dot{V}}_{O_{2}\text{peak}}$ improvement with fentanyl is similar to the ~17% improvement in ${\dot{V}}_{O_{2}\text{peak}}$ following exercise training in HFrEF patients (Smart & Marwick, 2004). These findings highlight the important role of locomotor muscle afferent feedback in the pathophysiology of exercise intolerance in patients with HFrEF.

**FIGURE 3:**
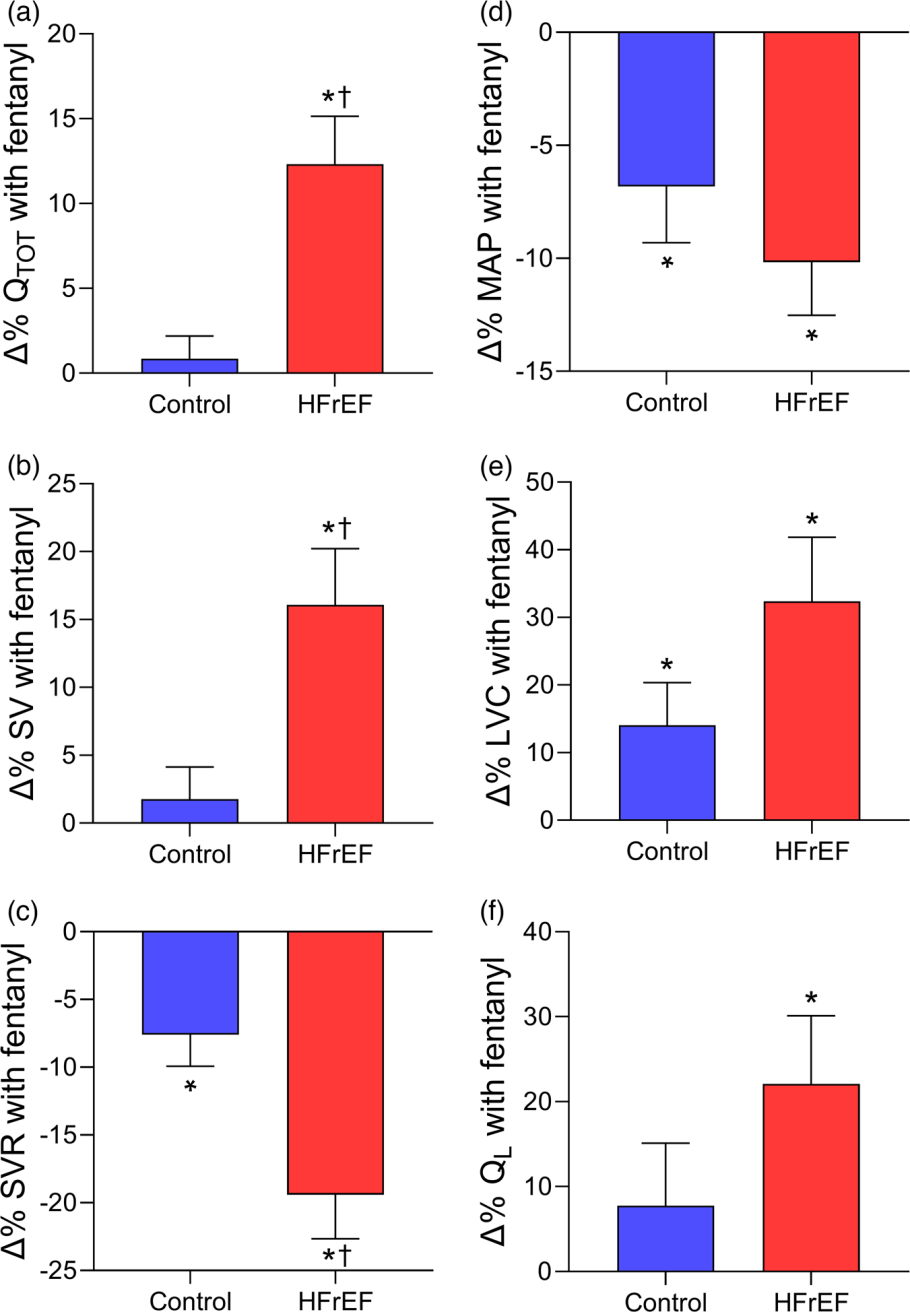
Changes in cardiovascular responses with lower lumbar intrathecal fentanyl compared to placebo at ${\dot{V}}_{O_{2}\text{peak}}$ in controls and HFrEF patients. Percentage changes in cardiac output (${\dot{Q}}_{TOT},a$), stroke volume (SV, b), systemic vascular resistance (SVR, c), mean arterial pressure (MAP, d), leg vascular conductance (LVC, e), and leg blood flow $\left({\dot{Q}}_{L},f\right)$ with fentanyl compared to placebo at ${\dot{V}}_{O_{2}\text{peak}}$ in healthy controls (blue bars) and HFrEF patients (red bars). Cardiac output, stroke volume and leg blood flow were higher with fentanyl than placebo in HFrEF patients. Controls and HFrEF patients had significant decreases in mean arterial pressure and systemic vascular resistance as well as increases in leg vascular conductance with fentanyl. The percentage change in cardiac output, stroke volume and systemic vascular resistance with fentanyl compared to placebo was greater in HFrEF than control. *Significantly different from placebo. †Significantly different from the healthy controls. Data used with permission and adapted from Smith, Joyner et al. (2020).

Recent studies have investigated the impact of these locomotor muscle afferents on cardiac output, locomotor muscle O_2_ delivery and ventilatory control during cycling and single-leg knee extensor exercise among patients with HFrEF (Amann et al., 2014; Olson et al., 2014; Smith, Joyner et al., 2020, Smith et al., 2022). These investigations demonstrated that, compared to the placebo condition, the increase in ${\dot{V}}_{O_{2}\text{peak}}$ with fentanyl in HFrEF patients was associated with greater cardiac output during maximal exercise via increased stroke volume (Smith, Joyner et al., 2020) (see Figure 3). During submaximal cycling exercise, stroke volume was higher and heart rate lower with fentanyl compared to placebo in HFrEF patients resulting in similar cardiac output responses between conditions (Smith, Joyner et al., 2020; Smith et al., 2022). These findings suggest that locomotor muscle afferents hinder the exercise-related increase in stroke volume particularly during maximal exercise among HFrEF patients.

In addition to these central haemodynamic changes during exercise under conditions that blunt group III/IV afferent feedback (fentanyl), changes in peripheral haemodynamics during exercise among HFrEF patients have also been studied. Leg vascular conductance, blood flow, ${\dot{V}}_{O_{2}}$ and O_2_ delivery were greater with fentanyl than placebo conditions in HFrEF patients during maximal exercise. In contrast, only leg vascular conductance was greater with fentanyl than placebo in healthy older control participants during maximal exercise (Smith, Joyner et al., 2020) (see Figure 3). During submaximal cycling exercise, blood pressure was lower with the fentanyl condition, but leg vascular conductance increased resulting in no overall effect on leg blood flow in HFrEF patients (Smith et al., 2022) – a similar result to that reported in older adults (Sidhu et al., 2015; Smith et al., 2022). During submaximal single-leg knee extensor exercise, Amann et al. found that the fentanyl condition reduced noradrenaline spillover, increased leg vascular conductance, and subsequently increased leg blood flow and O_2_ delivery in HFrEF patients (Amann et al., 2014). The discrepancy in peripheral haemodynamic responses with locomotor muscle afferent inhibition during submaximal exercise in HFrEF between these previous studies may be explained by limited competition among vascular beds during smaller muscle mass protocols (i.e., single-leg knee extensor) compared to whole body exercise.

Locomotor muscle afferents also impact ventilatory control during exercise in healthy adults and HFrEF patients (Amann et al., 2010; Olson et al., 2014; Smith, Hart et al., 2020, Smith, Joyner et al., 2020, 2022). Recent findings suggest that healthy ageing may lessen the contribution of the locomotor muscle afferents to the ventilatory response to exercise (Sidhu et al., 2015). During cycling exercise, locomotor muscle afferent inhibition via fentanyl has resulted in both a reduction and no change in ventilation in older adults (Olson et al., 2014; Smith, Hart et al., 2020, Smith, Joyner et al., 2020, 2022). The explanation for this discrepancy is unclear but may be due to the exercise protocol (incremental vs. steady state exercise) and the intensity of exercise. In HFrEF patients, locomotor muscle afferent inhibition via fentanyl has resulted in a reduced ventilatory response leading to an increased $P_{{aCO}_{2}}$ and decreased $P_{{aO}_{2}}$ across a range of submaximal exercise intensities and maximal exercise (Olson et al., 2014; Smith, Joyner et al., 2020, 2022). The reduction in ventilation and increase in $P_{{aCO}_{2}}$ with locomotor muscle afferent inhibition during exercise has been reported to be greater in HFrEF patients than older adults (Smith, Hart et al., 2020). Importantly, these previous studies have found no differences in the resting ventilatory responses to hypercapnia with fentanyl compared to placebo providing support that these ventilatory and cardiovascular responses discussed above are the result of inhibition of locomotor muscle group III/IV afferent feedback (and not cephalad migration of fentanyl to the brainstem) (Amann et al., 2010; Amann, Runnels et al., 2011, 2014; Olson et al., 2014; Smith, Joyner et al., 2020). The exaggerated ventilatory response in HFrEF patients during exercise (see Figure 1), in part from the locomotor muscle afferent feedback, has important implications for competition between respiratory and locomotor vasculature for cardiac output as discussed in detail below.

### 2.2 | Contribution of metabolically and mechanically sensitive afferents in HFrEF

**FIGURE 4:**
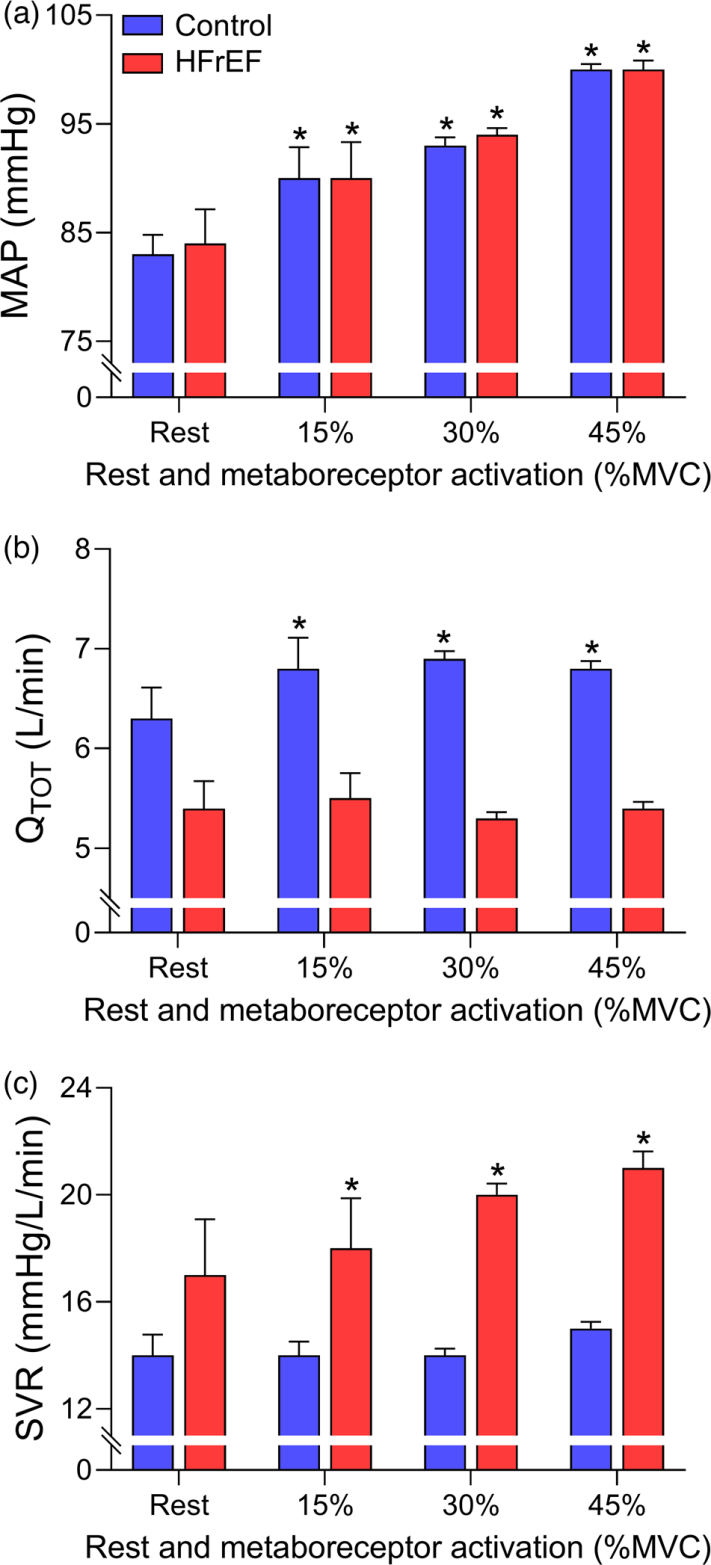
Cardiovascular responses to metaboreflex activation in controls and HFrEF patients. Mean arterial pressure (MAP, a), cardiac output (${\dot{Q}}_{\text{TOT}},\;b$), and systemic vascular resistance (SVR, c) with isolated metaboreflex activation (via post-exercise circulatory occlusion) following forearm exercise at 15%, 30% and 45% maximum voluntary contraction (MVC) in healthy controls (blue bars) and HFrEF patients (red bars). Controls and HFrEF patients had similar increases in mean arterial pressure with isolated metaboreflex activation. The controls increased mean arterial pressure via increases in cardiac output with metaboreflex activation. In contrast, HFrEF patients increased mean arterial pressure by increases in systemic vascular resistance. *Significantly different compared to rest. Data used with permission and adapted from Barrett-O’Keefe et al. 2018).

Both the metabolically and mechanically sensitive components of the exercise pressor reflex, commonly referred to as the metaboreflex and mechanoreflex, likely contribute to the abnormal autonomic and cardiovascular responses in HFrEF, although, the contribution of each component is debated (Middlekauff & Sinoway 2007; Piepoli & Coats, 2007). In humans, isolation of the metaboreflex (primarily via post-exercise circulatory occlusion following forearm exercise) has resulted in preserved and exaggerated blood pressure responses in HFrEF compared to healthy adults (with variable sympathetic nervous system activity responses reported) (Barrett-O’Keefe et al. 2018; Carrington et al., 2001; Crisafulli et al. 2007; Keller-Ross et al., 2014; Notarius et al., 2001; Piepoli et al. 1996, 1999; Shoemaker et al. 1998; Silber et al., 1998; Sterns et al., 1991). Despite these discrepancies, it is clear that the contribution of cardiac output and systemic vascular resistance to the metaboreflex-mediated blood pressure response is altered with the HF syndrome (Barrett-O’Keefe et al. 2018; Crisafulli et al., 2007; O’Leary et al., 2004). For example, Barrett-O’Keefe et al. reported the blood pressure, cardiac output, and systemic vascular resistance responses with isolated metaboreflex activation (via post-exercise circulatory occlusion) following forearm exercise at 15%, 30% and 45% maximum voluntary contraction (Barrett-O’Keefe et al., 2018) (see Figure 4). In the healthy adults, the increase in blood pressure with metaboreflex activation was due to increases in cardiac output. In HFrEF patients, metaboreflex activation resulted in similar increases in blood pressure compared to healthy adults at all contraction intensities. However, the increases in blood pressure in HFrEF patients during isolated metaboreflex activation was achieved by increases in systemic vascular resistance as opposed to changes in cardiac output and stroke volume, which contrasts with the responses in healthy adults. In HFrEF patients, isolation of the metaboreflex activation has been suggested to impair contractility and augment afterload contributing to the impairment of cardiac output and stroke volume responses (Barrett-O’Keefe et al., 2018; Crisafulli et al., 2007). The impact of metaboreflex activation on contractility and/or afterload provides important insights to the locomotor muscle afferent mediated impairment in stroke volume at maximal exercise intensities in HFrEF patients (Smith, Joyner et al., 2020).

Previous studies also provide support for exaggeration of the mechanically sensitive component of the exercise pressor reflex in HFrEF. In the rat infarct model of HFrEF, stimulation of the mechanoreflex via passive muscle stretch has consistently resulted in greater increases in sympathetic nerve activity and blood pressure compared to healthy rats (Koba et al., 2008; Li et al., 2004; Smith et al., 2005; Wang, LI et al., 2010). In human HFrEF, numerous methodologies have been used to stimulate the mechanoreflex, examples include static stretch, passive leg movement, and rhythmic involuntary muscle contractions. Similar to rat models of HFrEF, these studies in humans also provide support for an ‘overactive’ mechanoreflex in HFrEF patients (Ives et al., 2016; Middlekauff et al., 2001, 2004).

The mechanisms underlying the altered metabolically and mechanically sensitive components of the exercise pressor reflex in HFrEF are not well understood as recently reviewed (Grotle et al., 2020). The HF syndrome appears to modify the expression/density and sensitivity of the receptors and channels located on the group III and IV afferents (Grotle et al., 2020). To this point, recent studies have identified altered electrophysiological properties of channels and receptors in HFrEF associated with the exercise pressor reflex (Hong et al., 2021; Xing & Li, 2016). Future studies are needed to better understand the mechanisms underlying the HFrEF-mediated alterations to the mechanically and metabolically sensitive components of the exercise pressor reflex, particularly in humans with HFrEF.

In summary, locomotor muscle group III/IV afferent feedback plays an important role in the pathophysiology of exercise intolerance in the HF syndrome. Previous studies suggest that the locomotor muscle afferents in HFrEF impairs the central and peripheral haemodynamic responses during exercise, which directly contributes to the diminished exercise tolerance in these patients. To this point, these locomotor muscle afferents accelerate the development of peripheral fatigue in HF by impairing peripheral haemodynamic responses, but also facilitates the development of central fatigue (Weavil et al., 2021). In summary, locomotor muscle group III/IV afferent feedback plays an important role in the pathophysiology of exercise intolerance in the HF syndrome. Previous studies suggest that the locomotor muscle afferents in HFrEF impairs the central and peripheral haemodynamic responses during exercise, which directly contributes to the diminished exercise tolerance in these patients. To this point, these locomotor muscle afferents accelerate the development of peripheral fatigue in HF by impairing peripheral haemodynamic responses, but also facilitates the development of central fatigue (Weavil et al. 2021). It should be acknowledged that multiple components are involved in the neural control of the cardiovascular system in addition to locomotor muscle afferent feedback. Importantly, the HFrEF syndrome likely alters many of these components including central integration of the afferent feedback, efferent arm of the exercise pressor reflex, end-organ response (e.g., *α*-adrenergic receptor sensitivity, density, and/or receptor distribution), vascular transduction of sympathetic outflow, intramuscular bioenergetics and functional sympatholysis (Barrett-O’Keefe et al., 2019; Floras & Ponikowski, 2015; Nardone et al., 2022; Shenton & Pyner, 2016; Thomas et al., 2001; Wiener et al., 1986). It should be acknowledged that multiple components are involved in the neural control of the cardiovascular system in addition to locomotor muscle afferent feedback. Importantly, the HFrEF syndrome likely alters many of these components including central integration of the afferent feedback, efferent arm of the exercise pressor reflex, end-organ response (e.g., *α*-adrenergic receptor sensitivity, density, and/or receptor distribution), vascular transduction of sympathetic outflow, intramuscular bioenergetics and functional sympatholysis (BarrettO’Keefe et al., 2019; Floras & Ponikowski, 2015; Nardone et al., 2022; Shenton & Pyner, 2016; Thomas et al., 2001; Wiener et al., 1986).

## 3 | IMPACT OF RESPIRATORY MUSCLE WORK ON CARDIOVASCULAR RESPONSES IN HFrEF

The previous section described the abnormal locomotor muscle group III/IV afferent feedback mediated autonomic and cardiovascular responses in HFrEF; now we describe pulmonary system and respiratory muscle work abnormalities in HFrEF and their role in the pathophysiology of exercise intolerance in these patients. The HF syndrome is associated with pulmonary system and respiratory muscle alterations that are independent from smoking history. At rest, HFrEF patients have restrictive–obstructive lung patterns and lower lung diffusion capacity. HFrEF patients have a high prevalence of respiratory muscle weakness (i.e., ~43% of HFrEF patients have respiratory muscle strength <70% of predicted; Hamazaki et al., 2020) and lower respiratory muscle endurance (Walsh et al., 1996). HFrEF is associated with significant diaphragm maladaptation including fibre-type shift and atrophy, increased oxidative stress levels (likely contributing to diaphragmatic muscle atrophy and contractile dysfunction), and mitochondrial structural and functional abnormalities (Mangner et al., 2021). During exercise, HFrEF patients have reduced lung compliance, tachypnoeic breathing pattern (higher breathing frequency and smaller tidal volume) and an exaggerated ventilatory response (with the latter having high prognostic value, see Figure 1) (Arena et al., 2004; Cross et al., 2012; Dube et al., 2016; Kleber et al., 2000; Smith & Olson, 2019). Several factors contribute to these pulmonary abnormalities including pulmonary congestion (that has both direct and indirect effects on airway tone and/or sensitivity), cardiomegaly (augmented heart–lung interdependence) and weaker respiratory muscles. Further, mechanical constraints, mild ventilation–perfusion mismatch and elevated physiological dead space contribute to an exaggerated ventilatory response during exercise in HFrEF patients (Dube et al., 2016; Johnson et al., 2000; Olson et al., 2006; Smith & Olson, 2019; Sullivan et al., 1988). These pulmonary abnormalities result in altered respiratory mechanics and higher respiratory muscle work of breathing in HFrEF patients during exercise compared to healthy adults (Cross et al., 2012).

**FIGURE 5:**
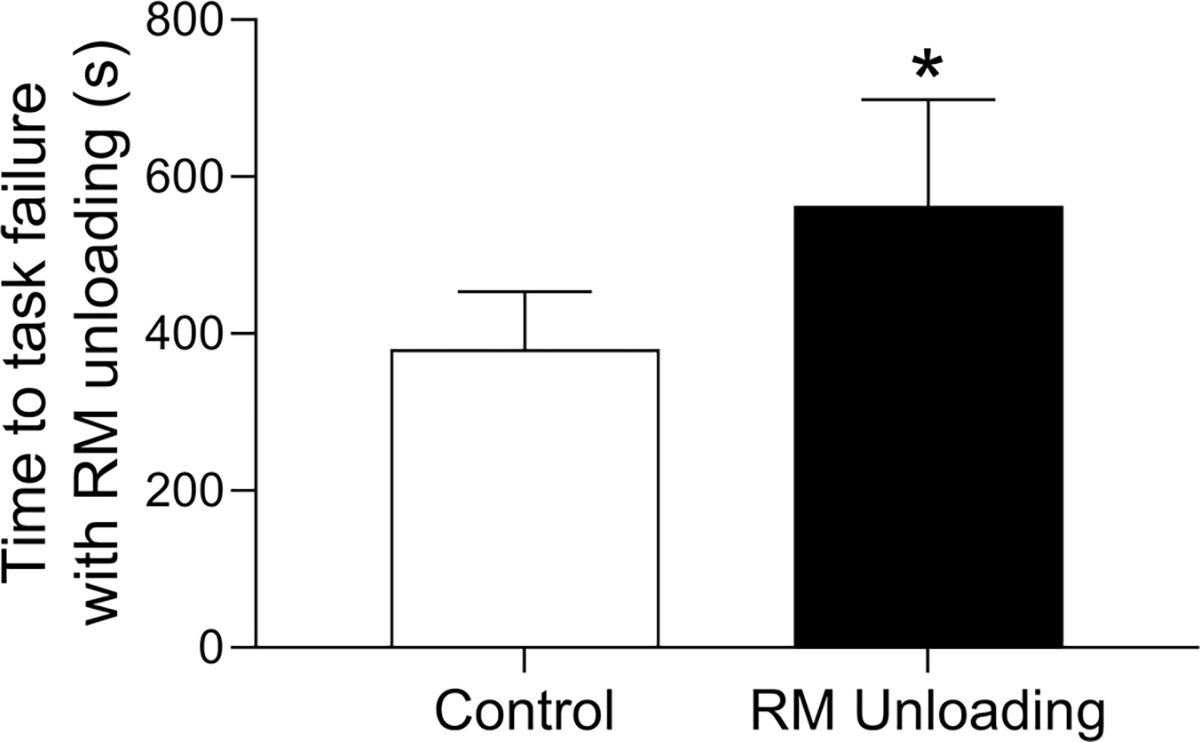
Impact of respiratory muscle unloading on submaximal exercise tolerance in HFrEF. In HFrEF patients, respiratory muscle unloading resulted in an ~50% increase in exercise time (i.e., time-to-task failure) during constant workload cycling at ~75% peak workload. *Significantly different from the control condition. Data used with permission and adapted from Borghi-Silva et al. (2008).

Respiratory muscle work influences exercise tolerance in healthy adults during high-intensity exercise. Specially, unloading the respiratory muscles results in increased time to exhaustion of ~14% during high-intensity exercise (i.e., ~90% ${\dot{V}}_{O_{2}\text{peak}}$ to exhaustion) (Harms et al., 2000). The elevated respiratory muscle work during exercise in HFrEF has important implications for exercise tolerance (Borghi-Silva et al., 2008; O’Donnell et al., 1999). For example, Borghi-Silva et al. investigated the impact of respiratory muscle unloading on time-to-task failure during constant workload cycling at ~75% peak workload in HFrEF patients (Borghi-Silva et al., 2008). Unloading the respiratory muscles resulted in a ~50% increase in exercise time (i.e., time-to-task failure) in these patients (see Figure 5). Previous studies provide support for the higher respiratory muscle work and altered respiratory mechanics during exercise in HFrEF impairing locomotor muscle O_2_ delivery and contributing to exercise intolerance for these patients. As such, we next discuss the influence of respiratory muscle work on stroke volume and locomotor muscle O_2_ delivery during exercise in HFrEF patients.

### 3.1 | Impact of intrathoracic pressure development on stroke volume in HFrEF

As the lungs and heart cohabitate within the thoracic cavity, intrathoracic pressure changes in response to ventilation influence stroke volume by impacting both ventricular filling and ejection (Cheyne et al., 2020). Specifically, negative intrathoracic pressure development lowers right atrial pressure, which increases venous return and ventricular transmural pressure during diastole thereby facilitating greater ventricular filling (Cheyne et al., 2020). This same increase in ventricular transmural pressure via negative intrathoracic pressure can increase left ventricular afterload and myocardial wall stress thereby impeding ventricular ejection during systole (Cheyne et al., 2020). Importantly, cardiac and pulmonary abnormalities associated with disease development will impact these cardiopulmonary interactions.

In healthy adult humans and canines, reducing the negative intrathoracic pressure during inspiration by 30–70% resulted in a 5–10% decrease in stroke volume during submaximal exercise and at ${\dot{V}}_{O_{2}\text{peak}}$ (Harms et al., 1998; Lalande et al., 2012; Miller et al., 2007; Olson et al., 2010). Importantly, these decreases in stroke volume occurred despite the venous return contributions from the skeletal muscle pump. Further, a greater degree of intrathoracic pressure reduction during inspiration appears to elicit a proportional decrease in stroke volume during exercise in healthy adults and canines (Lalande et al., 2012; Miller et al., 2007). In these studies, when the respiratory muscles are unloaded during inspiration, cardiac output is maintained due to increases in heart rate at submaximal exercise intensities (Lalande et al., 2012; Miller et al., 2007; Olson et al., 2010), but not during maximal exercise (Harms et al., 1998). The mechanisms by which intrathoracic pressure facilitates this increase in stroke volume during exercise have been suggested to include augmented venous return and increased ventricular transmural pressure facilitating greater ventricular filling (Lalande et al., 2012; Miller et al., 2007; Mitchell et al., 2005; Naughton et al., 1995). These findings suggest, in addition to the skeletal muscle pump, intrathoracic pressure development is necessary for the ‘normal’ stroke volume response during exercise in healthy adults.

**FIGURE 6:**
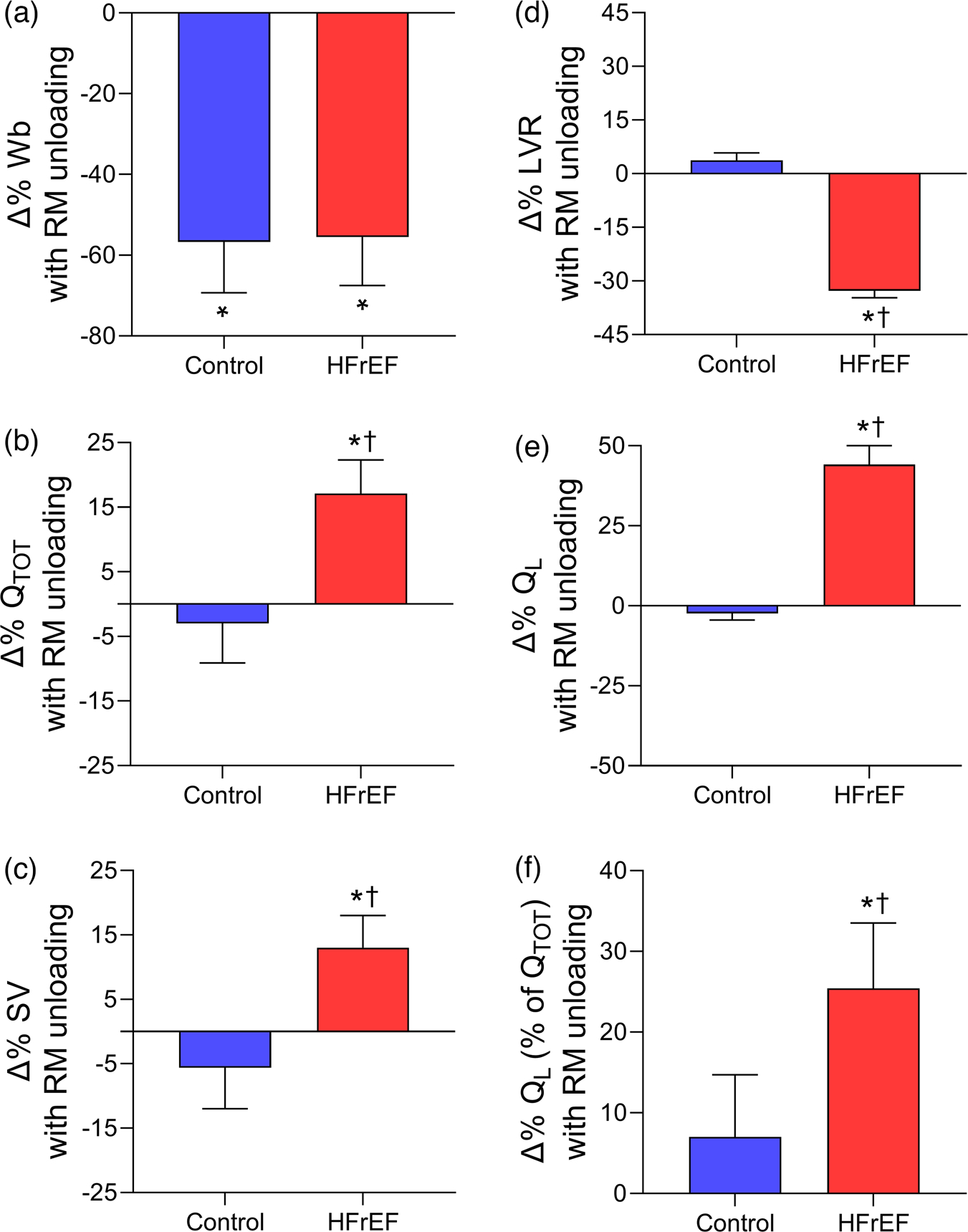
Changes in cardiovascular responses with respiratory muscle unloading during submaximal exercise in controls and HFrEF patients. Percentage changes in work of breathing ($W_{b},a$), cardiac output (${\dot{Q}}_{\text{TOT}},b$), stroke volume (SV, c), leg vascular resistance (LVR, d), leg blood flow (${\dot{Q}}_{L},e$), and leg blood flow (as a percentage of cardiac output) (f) with respiratory muscle unloading in healthy controls (blue bars) and HFrEF patients (red bars). Respiratory muscle unloading resulted in significant reductions in the work of breathing in controls and HFrEF patients. With respiratory muscle unloading, cardiac output, stroke volume, leg blood flow and leg blood flow (as a percentage of cardiac output) increased, and leg vascular resistance decreased in HFrEF patients. *Significantly different from the control condition. †Significantly different from the healthy controls. Data used with permission and adapted from Olson et al. (2010).

In contrast to healthy adults, intrathoracic pressure development during inspiration *impairs* stroke volume during exercise in HFrEF due to the cardiac and pulmonary abnormalities associated with the HF syndrome (Lalande et al., 2012; Miller et al., 2007; Olson et al., 2010). Specifically, Johnson and colleagues investigated the impact of intrathoracic pressure during inspiration on stroke volume during submaximal exercise in patients with HFrEF (Lalande et al., 2012; Olson et al., 2010). They found that unloading the respiratory muscles and reducing the negative intrathoracic pressure during inspiration by ~35–75% resulted in an ~5–13% increase in stroke volume during exercise in HFrEF patients (see Figure 6) (Lalande et al., 2012; Olson et al., 2010). Because heart rate was unaffected with respiratory muscle unloading in HFrEF patients (Lalande et al., 2012; Olson et al., 2010), the reduction in negative intrathoracic pressure during inspiration has resulted in increased cardiac output during exercise in HFrEF patients and canines (Miller et al., 2007; Olson et al., 2010). This finding is in direct contrast to the responses observed in healthy older adults. Further, the greater degree of intrathoracic pressure reduction was associated with proportional increases in stroke volume in HFrEF canines (Miller et al., 2007).

The intrathoracic pressure-mediated impairment of stroke volume in HFrEF has been proposed to be due to direct ventricular interaction as well as increased left ventricular transmural pressure gradient hindering left ventricular ejection (Cheyne et al., 2020; Genovese et al., 1995; Lalande et al., 2012; Miller et al., 2007). Taken together, the intrathoracic pressure during inspiration is necessary for the ‘normal’ stroke volume response in healthy adults during exercise. In striking contrast, the intrathoracic pressure during inspiration impairs the stroke volume response during submaximal exercise in HFrEF, which has important implications for O_2_ delivery and exercise intolerance in these patients.

### 3.2 | Impact of respiratory muscle work on locomotor muscle O_2_ delivery in HFrEF

During high-intensity exercise, the metabolic cost of breathing represents 10–15% of total ${\dot{V}}_{O_{2}}$ in healthy adults (Aaron et al., 1992). The high metabolic cost associated with the respiratory muscles during high-intensity exercise results in physiological competition for cardiac output between the respiratory and locomotor muscles in healthy adults. Studies investigating this notion have experimentally unloaded the respiratory muscles during exercise and measured the locomotor muscle blood flow responses (Miller et al., 2007; Olson et al., 2010; Wetter et al., 1999). In healthy adults, reducing the work of breathing by ~63% during maximal exercise cycling led to a ~5% increase in leg blood flow (Harms et al., 1997). The increase in leg blood flow during maximal exercise with inspiratory muscle unloading was primarily due to attenuated leg sympathetic nerve activity and increased leg vascular conductance. As noted above, unloading the inspiratory muscles resulted in a ~9% decrease in cardiac output during maximal exercise (Harms et al., 1998). During lower intensity exercise (~50–75%). The high metabolic cost associated with the respiratory muscles during high-intensity exercise results in physiological competition for cardiac output between the respiratory and locomotor muscles in healthy adults. Studies investigating this notion have experimentally unloaded the respiratory muscles during exercise and measured the locomotor muscle blood flow responses (Miller et al., 2007; Olson et al., 2010; Wetter et al., 1999). In healthy adults, reducing the work of breathing by ~63% during maximal exercise cycling led to a ~5% increase in leg blood flow (Harms et al., 1997). The increase in leg blood flow during maximal exercise with inspiratory muscle unloading was primarily due to attenuated leg sympathetic nerve activity and increased leg vascular conductance. As noted above, unloading the inspiratory muscles resulted in a ~9% decrease in cardiac output during maximal exercise (Harms et al., 1998). During lower intensity exercise (~50–75% ${\dot{V}}_{O_{2}\text{peak}}$), unloading the inspiratory muscles has led to decreases in muscle sympathetic nerve activity in healthy adults, but no changes in leg blood flow likely due to a cardiac output reserve at these submaximal intensities (Dominelli et al., 2019; Wetter et al., 1999).

As noted above, the HF syndrome is associated with reduced respiratory muscle strength and endurance as well as diaphragm muscle maladaptation. Additionally, HFrEF patients have an exaggerated ventilatory response during exercise coupled with a higher work of breathing for a given ventilation (compared to healthy adults) (see Figure 1) (Cross et al., 2012; Dube et al., 2016). The higher work of breathing results in exaggerated respiratory muscle oxygen cost and blood flow requirement during exercise in HFrEF. HFrEF patients can also exhibit an impaired cardiac output response to submaximal exercise and minimal cardiac reserve. In this context, the elevated metabolic cost of the respiratory muscles in HFrEF leads to physiological competition for cardiac output between the respiratory and locomotor muscles during submaximal exercise (i.e., exercise intensities analogous to activities of daily living), and this competition for cardiac output contributes to exercise intolerance. Specifically, reducing the work of breathing by 55% in HFrEF patients during submaximal exercise resulted in a 44% increase in leg blood flow, while leg blood flow was not altered with respiratory muscle unloading in healthy older adults (Olson et al., 2010) (see Figure 6).

Several mechanisms likely contribute to the increase in leg blood flow during exercise in HFrEF patients with respiratory muscle unloading. First, unloading the respiratory muscles during exercise in HFrEF patients resulted in a 17% increase in cardiac output (via increases in stroke volume as discussed above) that substantially contributed to the increase in leg blood flow (Olson et al., 2010). Second, the increase in leg blood flow with respiratory muscle unloading was due to increases in leg vascular conductance during exercise (see Figure 6). Importantly, the increase in leg blood flow with respiratory muscle unloading in HFrEF was disproportionately greater than the increase in cardiac output (and presumably from the respiratory muscles whose work, and therefore oxygen demands, were lower). Taken together, these findings indicate that respiratory muscle work contributes to exercise intolerance by reducing locomotor muscle blood flow during submaximal exercise in HFrEF by impairing stroke volume as well as heightening sympathetic outflow thereby increasing locomotor muscle vascular resistance.

The primary mechanism proposed to be responsible for the respiratory muscle work mediated sympathetic vasoconstriction and resultant reduced locomotor muscle blood flow in HFrEF is a metaboreflex arising from the respiratory muscles (‘respiratory muscle metaboreflex’). Evidence supporting the respiratory muscle metaboreflex comes from both animal and human studies. Specifically, fatiguing contractions of the diaphragm elicit increases in type IV (primarily metabolically sensitive) afferent discharge in the anaesthetized rat (Hill, 2000). Further, lactic acid infusion into the phrenic circulation decreases limb blood flow via sympathetically mediated vasoconstriction at rest and during exercise in canines (Rodman et al., 2003). In otherwise resting healthy adults, high respiratory muscle work and the concomitant accumulation of metabolites activates the respiratory muscle metaboreflex resulting in time-dependent increases in sympathetic nerve activity, leg vasoconstriction, blood pressure and subsequently decreases in leg blood flow (Sheel et al., 2001, 2002; Smith et al., 2016; Smith, Alexander et al., 2017; St Croix et al., 2000). Importantly, the HF syndrome is associated with abnormal cardiovascular responses with respiratory muscle metaboreflex activation. For example, patients with HFrEF have greater increases in limb vasoconstriction and decreases in limb blood flow compared to healthy adults when the respiratory muscle metaboreflex is activated by performing high respiratory muscle work to task failure (Chiappa et al., 2008). These findings suggest that an abnormal respiratory muscle metaboreflex in HFrEF contributes to impairment in locomotor muscle O_2_ delivery during exercise in these patients.

In summary, HFrEF patients have pulmonary system and respiratory muscle abnormalities that contribute to elevated respiratory muscle work during exercise. This high respiratory muscle work and associated high metabolic cost in HFrEF significantly contributes to exercise intolerance in these patients. Previous studies indicate that the respiratory muscle work in HFrEF impairs locomotor muscle O_2_ delivery through its impact on stroke volume and sympathetic vasoconstriction of the locomotor muscles.

## 4 | POTENTIAL CONSEQUENCES OF LOCOMOTOR MUSCLE AFFERENT FEEDBACK AND RESPIRATORY MUSCLE WORK DURING EXERCISE IN HFrEF

**FIGURE 7:**
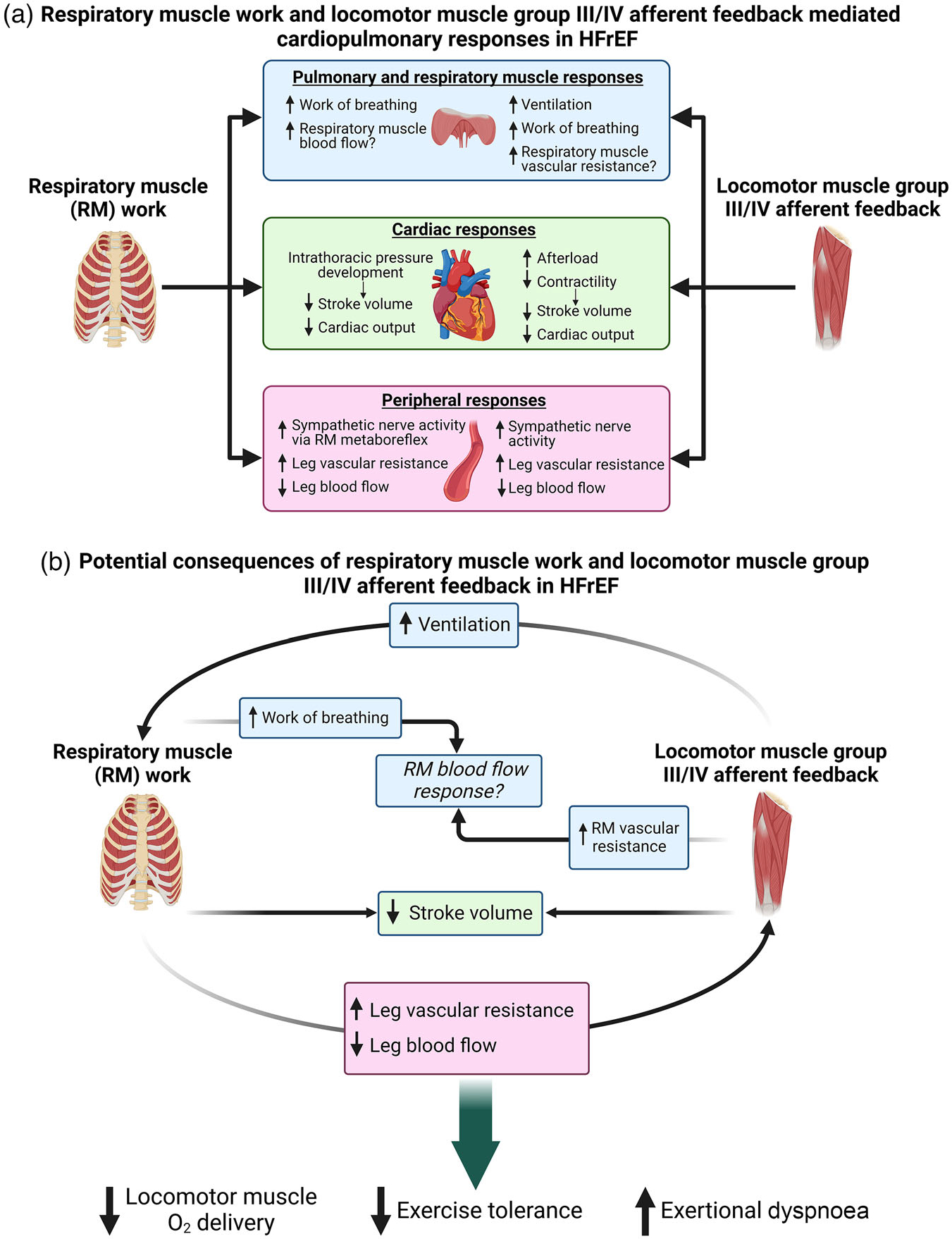
Potential consequences between locomotor muscle afferent feedback and respiratory muscle work in HFrEF during exercise. (a) Summary of the locomotor muscle group III/IV afferent feedback and respiratory muscle work mediated abnormal autonomic and cardiopulmonary responses in HFrEF patients during exercise outlined in this review. (b) Illustration of potential autonomic and cardiopulmonary interactions between locomotor muscle afferent feedback and respiratory muscle work in HFrEF, and the subsequent impact on locomotor muscle O_2_ delivery and exercise tolerance.

Figure 7a shows a diagrammatic summary of the locomotor muscle group III/IV afferent feedback and respiratory muscle work-mediated abnormal autonomic and cardiopulmonary responses in HFrEF patients during exercise. Figure 7b illustrates potential autonomic and cardiopulmonary consequences of locomotor muscle afferent feedback and respiratory muscle work in HFrEF. We propose that the concomitant effects of the locomotor muscle group III/IV afferents and high respiratory muscle work worsen exercise tolerance and exertional symptomology for HFrEF patients. Unloading the respiratory muscles during exercise results in decreased leg exertional discomfort in HFrEF patients (Borghi-Silva et al., 2008; O’Donnell et al., 1999). Additionally, it has been shown that the improvement in exercise tolerance with respiratory muscle unloading is associated with the degree of leg exertional discomfort reduction in these patients O’Donnell et al. (1999). Mechanistically, respiratory muscle work impacts locomotor muscle O_2_ delivery during exercise in HFrEF via the effects of negative intrathoracic pressure generation on stroke volume and respiratory muscle-mediated vasoconstriction of locomotor muscle vascular beds. It would be expected that the constraint of locomotor muscle O_2_ delivery by high respiratory muscle work would increase both the reliance on anaerobic energy sources and accumulation of metabolic byproducts. This would subsequently result in higher levels of locomotor muscle group III/IV reflex activation further elevating the ventilatory response as well as hastening the development of locomotor muscle fatigue.

Altered locomotor muscle group III/IV afferent feedback in HFrEF patients also has important implications for respiratory muscle work. Increases in sympathetic nervous system activation evoked by locomotor muscle afferent feedback impacts respiratory muscle blood flow regulation when combined with high locomotor muscle metabolic demand during exercise (Sheel et al., 2018). In healthy adults, Sheel et al. have proposed that metaboreflex-mediated increases in sympathetic nervous activity from the locomotor muscles hinder O_2_ delivery to the respiratory muscles during high-intensity exercise and vice versa (Sheel et al., 2018). In the setting of cardiac dysfunction along with pulmonary system/respiratory muscle abnormalities and heightened sympathetic nerve activity in HFrEF, respiratory muscle vascular resistance arising from locomotor muscle afferent feedback (and the exercise pressor reflex) will likely be accentuated. In HFrEF patients, this will likely result in a vicious cycle between the substantially high respiratory muscle work (and metabolic cost) and locomotor muscle afferent feedback-mediated respiratory muscle vascular resistance in regard to respiratory muscle blood flow during exercise. This vicious cycle between respiratory muscle work and locomotor muscle afferent feedback has important implications for exercise intolerance and exertional dyspnoea in HFrEF. To date, the impact of respiratory muscle work and locomotor muscle afferent feedback on respiratory muscle blood flow in HFrEF patients is largely unclear. In the rat infarct model of HFrEF, diaphragm muscle blood flow is elevated (in proportion to lung water weight, suggestive of respiratory muscle work), while locomotor muscle blood flow is reduced during submaximal exercise compared to healthy rats (Musch, 1993; Smith, Hageman et al., 2017). More recently, the impact of locomotor muscle work on respiratory muscle blood flow has been investigated in chronic obstructive pulmonary disease (COPD) patients. Locomotor muscle work has been reported to impair intercostal, scalene and rectus abdominis blood flow responses during high intensity cycling exercise in patients with COPD (Louvaris et al., 2021). Future studies are necessary to elucidate how locomotor muscle work, and the locomotor muscle afferents in particular, affect respiratory muscle blood flow regulation in patients with HFrEF.

The locomotor muscle group III/IV afferents contribute to the exaggerated ventilatory response in HFrEF during exercise (Olson et al., 2014; Smith et al., 2022). In fact, locomotor muscle afferent inhibition via intrathecal fentanyl reduces the ventilatory response in HFrEF patients and may ‘normalize’ the ventilatory response (i.e., the ventilatory response with fentanyl in HFrEF is similar to healthy adults with placebo) (Smith et al., 2022). In the context of the respiratory muscle work and abnormal respiratory metaboreflex in HFrEF, the impact of locomotor muscle afferents on the ventilatory response has two important implications. First, the locomotor muscle afferent-mediated increase in ventilation in HFrEF is primarily due to increases in breathing frequency. This higher breathing frequency translates into higher inspiratory flow rates and likely more negative intrathoracic pressure generation required to achieve the ventilatory response (and breathing strategy) during exercise. This is important for HFrEF patients as the negative intrathoracic pressure generation with inspiration is negatively related to stroke volume during exercise. Second, the locomotor muscle afferent-mediated increase in ventilation will heighten the work and oxygen cost of breathing during exercise in HFrEF patients. This higher respiratory muscle work may elicit commensurate levels of locomotor muscle vasoconstriction. To this point, the reduction in the work of breathing with respiratory muscle unloading during exercise in HFrEF was reported to be proportional to the increase in leg blood flow (as a percentage of cardiac output) (Olson et al., 2010). Taken together, the HF syndrome is associated with abnormal autonomic and cardiovascular responses arising from the locomotor muscle group III/IV afferent feedback and respiratory muscle work, and the concomitant effects likely worsen exercise tolerance and exacerbate exertional symptomology in HFrEF patients.

## 5 | SUMMARY AND FUTURE DIRECTIONS

In summary, locomotor muscle group III/IV afferent feedback and respiratory muscle work play an important role in the pathophysiology of exercise intolerance in the HF syndrome. The locomotor muscle afferent feedback in HFrEF results in impaired central and peripheral haemodynamic responses as well as augmenting the ventilatory response during exercise directly contributing to the diminished exercise tolerance in these patients. The high respiratory muscle work in HFrEF significantly contributes to exercise intolerance by impairing locomotor muscle O_2_ delivery. Further, there is direct and indirect evidence that the concomitant effects of the locomotor muscle afferent feedback and high respiratory muscle work worsen exercise tolerance and exertional symptomology for HFrEF patients.

There are numerous future directions that need to be pursued to elucidate these mechanisms and potential therapeutic strategies to mitigate these abnormal responses in HF. It is important to directly investigate the proposed consequences arising from the locomotor muscle afferent feedback and respiratory muscle work in HF during exercise (see Figure 7). This will likely rely on innovative experimental designs and novel methodologies using both animal models and human HF to tease out these mechanisms. The arterial baroreflex modulates blood pressure (primarily via systematic vascular resistance) and interacts with the skeletal muscle metaboreflex (Ichinose et al., 2002). In HFrEF, the baroreflex is altered with diminished ability to buffer the increase in sympathetic nervous activity and systemic vascular resistance arising from metaboreflex activation in a pacing-induced canine model of HFrEF (Kaur et al., 2018; Kim et al., 2005). Understanding the role the baroreflex plays in modulating the autonomic and cardiovascular responses arising from locomotor muscle afferent feedback and respiratory muscle work (as well as respiratory muscle blood flow responses) during exercise in HFrEF patients is critical. It is important to note that this review has focused on HFrEF, but ~50% of HF patients are diagnosed with HF with preserved ejection fraction (HFpEF) (Tsao et al., 2022). A recent study has reported that HFpEF patients have abnormal cardiovascular responses to isolated metaboreflex activation via post-exercise circulatory occlusion following forearm exercise compared with controls (Roberto et al., 2017). Recent studies have also found respiratory muscle and pulmonary system abnormalities in HFpEF patients including lower inspiratory muscle strength (which was related to higher exertional dyspnoea during low-intensity exercise), lower inspiratory muscle endurance, exaggerated cardiovascular responses to inspiratory muscle metaboreflex activation and higher work of breathing during exercise (Hammer et al., 2022; Villarraga et al., 2023; Warner et al., 2023). Future research is needed to better understand how HFpEF impacts the autonomic and cardiovascular responses arising from locomotor muscle afferent feedback and respiratory muscle work during exercise. Lastly, we must identify clinically relevant interventions that mitigate the abnormal autonomic and cardiovascular responses arising from locomotor muscle reflexes and respiratory muscle work. Preliminary evidence support a beneficial role for exercise training and inspiratory muscle training to ameliorate these abnormal mechanisms in HF patients (e.g., Antunes-Correa et al., 2014; Chiappa et al., 2008; Piepoli et al., 1996; Smith & Taylor, 2022; Wang, Pan et al., 2010; Winkelmann et al., 2009). However, there is a clear need to understand how these and other innovative interventions mechanistically impact the locomotor and respiratory muscle abnormalities that contribute to exercise intolerance and exertional dyspnoea in patients with HF. In addition, it would be of high value to investigate innovative exercise prescription strategies such as one-legged exercise and whole-body exercise with respiratory muscle unloading (e.g., via heliox) on locomotor and respiratory muscle abnormalities exhibited by patients with HF.

## Highlights

### What is the topic of this review?

This review is focused on understanding the role that locomotor muscle group III/IV afferent feedback and respiratory muscle work play in the pathophysiology of exercise intolerance in patients with heart failure.

### What advances does it highlight?

This review proposes that the concomitant effects of locomotor muscle afferent feedback and respiratory muscle work worsen exercise tolerance and exacerbate exertional dyspnoea in patients with heart failure.
